# Propionyl-l-carnitine mitigates ischemia-reperfusion injury in rat epigastric island flaps

**DOI:** 10.1016/j.heliyon.2024.e27448

**Published:** 2024-03-01

**Authors:** Atilla Adnan Eyuboglu, Ovunc Akdemir, Oytun Erbas, Mustafa Tonguc Isken, Feng Zhang, William C. Lineaweaver

**Affiliations:** a Arel University, Department of Plastic, Reconstructive and Aesthetic Surgery, Istanbul, Turkey; b Aydin University, Department of Plastic, Reconstructive and Aesthetic Surgery, Istanbul, Turkey; c Bilim University, Department of Physiopathology, Istanbul, Turkey; d Bahcesehir Medical University, Plastic, Reconstructive and Aesthetic Surgery, Istanbul, Turkey; e PhD University of Mississippi Medical Center, Division of Plastic Surgery, Microsurgery, 2500 North State Street, Jackson, MS 39216, USA; f Vanderbilt Bill Wilkerson Center for Otolaryngology and Communication Sciences, Plastic, Reconstructive and Aesthetic Surgery, Nashville, TN, USA

**Keywords:** Carnitine, Flap survival, Inflammation, Ischemia-reperfusion injury, Necrosis, Oxidative stress

## Abstract

**Background:**

Ischemia-reperfusion injury presents a substantial concern in various medical scenarios, notably in reconstructive surgery involving tissue flaps. Despite reports on the protective benefits of Propionyl-l-carnitine against ischemia-reperfusion injury, a thorough assessment of its efficacy in epigastric island flap models is currently lacking.

**Methods:**

Sixteen male Sprague-Dawley rats underwent epigastric island flap surgery and were divided into two groups: a Propionyl-l-carnitine group that received intraperitoneal Propionyl-l-carnitine prior to ischemia induction and a sham group that received saline treatment. A comprehensive evaluation was performed including macroscopic, biochemical and histological assessments encompassing measurements of flap survival areas, lipid peroxidation (malondialdehyde), glutathione, myeloperoxidase, nitric oxide and peripheral neutrophil counts.

**Results:**

The Propionyl-l-carnitine group demonstrated significantly increased flap survival areas when compared to the sham group. Administration of Propionyl-l-carnitine led to reduced malondialdehyde levels and elevated glutathione levels indicating a reduction in oxidative stress. Furthermore, the Propionyl-l-carnitine group exhibited lower myeloperoxidase levels, higher nitric oxide levels and reduced peripheral neutrophil counts, suggesting a decrease in the inflammatory response. Histopathological analysis revealed decreased levels of inflammation, necrosis, polymorphonuclear leukocyte infiltration and edema in the Propionyl-l-carnitine group. Additionally, vascularity was enhanced in the Propionyl-l-carnitine group.

**Conclusion:**

This study provides compelling evidence that Propionyl-l-carnitine administration effectively mitigates the deleterious effects of ischemia-reperfusion injury in epigastric island flaps. This is substantiated by the improved flap survival, diminished oxidative stress and inflammation, as well as the enhanced vascularity observed. Propionyl-l-carnitine emerges as a promising therapeutic intervention to enhance tissue flap survival in reconstructive surgery, warranting further exploration through larger-scale investigations.

## Highlights

• Propionyl-l-carnitine administration mitigates the damaging effects of I–R injury in epigastric island flaps, as evidenced by improved flap survival, reduced oxidative stress, inflammation, and enhanced vascularity.
• Propionyl-l-carnitine holds promise as a therapeutic intervention to enhance tissue flap survival in reconstructive surgery.
• Propionyl-l-carnitine administration reduced lipid peroxidation levels and elevated glutathione levels.
• Histopathological analysis showed decreased inflammation, necrosis, leukocyte infiltration, and edema with Propionyl-L-carnitine.

## 1 Introduction

Ischemia-reperfusion injury in skin/muscle flaps can lead to partial or complete flap loss without an apparent cause. This injury is a multifaceted problem involving apoptosis, the generation of oxygen radicals, platelet aggregation and interactions between leukocytes and endothelium. It arises from the acute interruption of blood flow within the microvasculature. Recent attention has centered on the interplay between neutrophils and endothelial cells in the context of ischemia-reperfusion injury (I/R) [1]. Ischemia-reperfusion injury plays a pivotal role in a wide range of diseases and can lead to exacerbated damage during surgical procedures. While considerable research efforts have been dedicated to alleviating its effects on brain, liver, kidney and cardiac tissues, the examination of its implications in the context of skeletal muscle has not received commensurate attention [2]. Ischemia-reperfusion injury following revascularization is an inherent pathophysiological process that occurs regardless of a surgeon's level of expertise [3].

Propionyl-l-carnitine (PLC), alternatively known as β-trimethylamino-β-hydroxybutyrate, is a naturally occurring compound present in its unesterified form within mammalian cells. It exists in various derivatives such as acetyl-l-carnitine, propionyl-l-carnitine and palmitoyl-l-carnitine. These derivatives are sourced from dietary components primarily found in meat and dairy products and are also synthesized endogenously, particularly within the liver and kidney [4]. Propionyl-l-carnitine's primary function is to serve as a co-transporter aiding in the translocation of long-chain free fatty acids from the cytosol into the mitochondria. Inside the mitochondria, these fatty acids are harnessed for β-oxidation leading to the production of acetyl coenzyme A as part of the tricarboxylic acid cycle. This metabolic process culminates in the generation of Adenosine Triphosphate (ATP) through the electron transport chain and oxidative phosphorylation. However, it should be noted that this process consumes a substantial amount of oxygen. In the end, oxygen is converted to water and the formation of reactive oxygen species (ROS) is curtailed [5]. Moreover, propionyl-l-carnitine demonstrates its antioxidant characteristics by boosting the function of key antioxidant enzymes including glutathione peroxidase (GSH), catalase and superoxide dismutase (SOD). It also has the capacity to bind to metal ions that play a role in generating ROS, such as ferrous ions. In the realm of antioxidant activity, propionyl-l-carnitine shares similarities with renowned antioxidants like alpha-tocopherol [6]. Although the precise mechanisms and advantages of PLC and its derivatives in the human body remain partially elucidated, it is established that tissues with elevated energy requirements such as skeletal and cardiac muscles, possess a notable presence of PLC. The protective attributes of PLC and its derivatives against I/R are predominantly attributed to their antioxidant properties. By restraining the generation of ROS during aerobic metabolism, they can ameliorate the harm resulting from I/R injury [7].

To contribute to the understanding of this subject, we utilized PLC in an ischemia-reperfusion injury model employing epigastric island flaps in rats, similar to our previous investigation into the effects of Trimetazidine on reperfusion injury [8]. In the light of aforementioned information from the literature, We have examined the effects of PLC on reperfusion damages using the skin flap model in rats.

## 2 Materials and methods

The biostatistics department determined the number of subjects, aiming to minimize the required number of animal groups while ensuring statistical significance. Sixteen male Sprague-Dawley rats, weighing between 230 and 280 g, were enrolled in this study. The experimental procedures followed the ethical guidelines set forth by the National Research Council for the care and utilization of laboratory animals. The reduction of the upper and lower incisor teeth was executed using a motor tool (Electric Variable Speed Motor Tool, Model 380–5, Dremel Co. Racine, WI, lowest speed) equipped with an abrasive cutting disc following the surgical procedure, as a preventative measure against auto cannibalism.

## 3 Experimental protocol

### 3.1 The characteristics of the experimental protocol were as follows

• The animals were divided into two groups, each comprising eight rats.
• In the PLC group, the flap was elevated following intraperitoneal administration of Propionyl-l-carnitine, after which ischemia was induced.
• In the sham group, the flap was elevated after intraperitoneal application of saline, and ischemia was subsequently induced.
• Biochemical samples were collected 24 h after reperfusion for analysis.
• On the 10th postoperative day, the rats were euthanized, and the flaps were photographed to assess flap survival. Additionally, tissue samples were collected for histopathological examination.

### 3.2 Surgical procedures

**Fig. 1:**
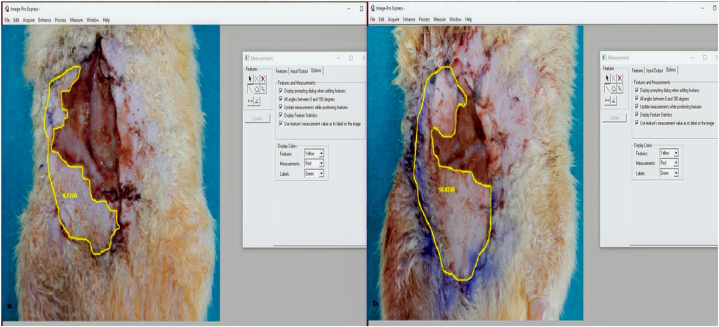
The surviving flap area was determined using Image-Pro Express program (Version 6.0, Media Cybernetics, USA). (a) Represents the Sham group, and (b) the Carnitine group.

All surgical procedures were conducted by the same surgeon (OA). Following intramuscular administration of xylazine (15 mg/kg; Alfazyne®, Alfasan International BV, Netherlands) and ketamine (50 mg/kg; Ketalar®, Pfizer Warner-Lambert, Turkey). The abdominal and inguinal regions of the rats were carefully shaved. Aseptic conditions were maintained using cetrimide and chlorhexidine gluconate (Savlon®, GlaxoSmithKline, Brentford, UK). Subsequently, the animals were positioned in the supine posture on the operating table. A 6 × 4 cm^2^ epigastric flap was meticulously marked and delineated using a skin marker as described on our previous studies [8,9]. In the PLC group (group 2), intraperitoneal administration of PLC (100 mg/kg body weight in 0.5 mL of saline) from Sigma Tau Co., Pomezia (Italy) was carried out. Approximately 30 min following the PLC injection, the flap, encompassing the subcutaneous and superficial fascia at the distal section of the inguinal ligament was raised in a craniocaudal direction. Subsequently, the epigastric artery and vein were meticulously identified and separated from the surrounding tissue. To secure the pedicle, an Acland V2 micro clamp was applied for a duration of 10 h (Fig. 1). (see

**Fig. 2:**
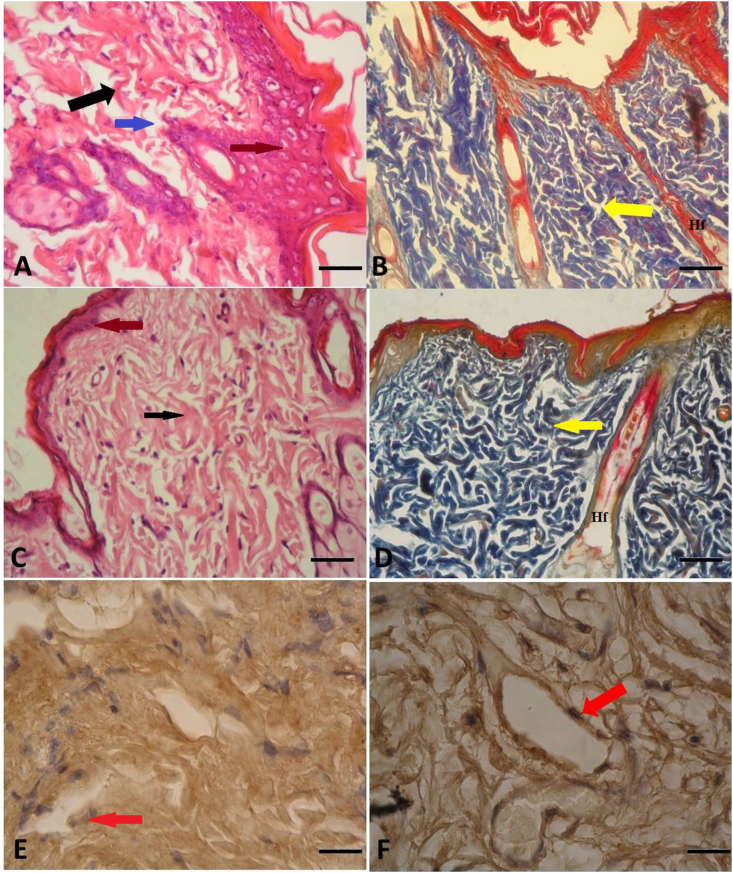
Histopathological examination of two experimental groups. (A) Displays the histopathological view of the Sham group with hematoxylin and eosin (HE) staining, original magnification × 10. (Black arrow indicates discolored collagen bundles; brown arrow indicates polymorphonuclear leukocyte [PMNL] infiltration; blue arrow indicates edema.) (B) Shows the Sham group histopathological view with Mallory Azan (MA) staining, original magnification × 10. (Yellow arrow points to discolored collagen bundles, and ‘Hf’ indicates degenerated hair follicle.) (C) Depicts the Carnitine group histopathological view with HE staining, original magnification × 10. (Black arrow signifies a significant difference from the sham group in collagen structure, while the brown arrow indicates significantly reduced PMNL infiltration compared to the sham group.) (D) Presents the Carnitine group histopathological view with MA staining, original magnification × 10. (Yellow arrow signifies a significant difference from the sham group in collagen structure, and ‘Hf’ represents a significant difference from the sham group in hair follicle degeneration.) (E) Shows anti-inducible nitric oxide synthase (iNOS) staining in the Sham group, original magnification × 40. (Red arrow points to anti-iNOS [+] endothelial cells.) (F) Displays anti-iNOS staining in the Carnitine group, original magnification × 40. (Red arrow again indicates anti-iNOS [+] endothelial cells.). (For interpretation of the references to color in this figure legend, the reader is referred to the Web version of this article.)

Following a 10-h period, the clamp was removed, and arterial-venous flow was reestablished using peristalsis. The flap exhibited an increase in temperature and a change in color before being repositioned and sutured with 4–0 polypropylene sutures. In the sham group (group 1), only 0.5 mL of physiological serum was injected 30 min prior to reperfusion via the saphenous vein. The flap in this group remained untreated. Under general anesthesia and with intramuscular ketamine and xylazine for analgesia, 1 × 1 cm^2^ tissue samples were obtained from the same sides of all flaps 24 h after reperfusion for subsequent biochemical analysis. After a period of 10 days, the rats were euthanized using a lethal dose of ketamine and images were captured to quantify the surviving area of the flap. To ensure the integrity of the necrotic region, 1 × 1 cm^2^ samples were extracted from the same sides of all flaps after the 10th-day images were taken for histological assessments.

### 3.3 Macroscopic measurement of the surviving flap area

Seven days after the operation, photographs of the flaps were taken using a Canon EOS 600 d camera positioned at a 25 cm distance. The surviving flap area was subsequently computed using the Image-Pro Express program (Version 6.0, Media Cybernetics, USA). To obtain this value, the necrotic area was deducted from the overall flap area [ [9]].

### 3.4 Histopathological and biochemical analysis

Lipid peroxidation, tissue glutathione, nitric oxide, myeloperoxidase levels and peripheral neutrophil counts were analyzed.

### 3.5 Measurement of tissues lipid peroxidation (MDA)

Lipid peroxidation was assessed in tissue specimens, each weighing approximately 100 mg, through the measurement of MDA levels as thiobarbituric acid reactive substances (TBARS). In brief, trichloroacetic acid and the TBARS reagent were introduced to the tissue samples, creating a mixture that was subsequently incubated at 100 °C for a duration of 60 min. After a period of cooling in an ice bath, the samples underwent centrifugation at 3000 rpm for 20 min and the absorbance of the resulting supernatant was recorded at 535 nm. MDA levels were quantified using a standard calibration curve generated with tetraethoxypropane and the values were expressed in nanomoles per gram of protein [10].

### 3.6 Measurement of tissue glutathione (GSH) level

The determination of the GSH content in tissue samples, each weighing approximately 100 mg was accomplished spectrophotometrically employing Ellman's method. In this assay, thiols within the samples reacted with 5,5′-dithiobis-(2-nitrobenzoic acid) (DTNB), resulting in the formation of a colored anion with a maximum peak absorbance at 412 nm. The GSH levels were quantified via a standard calibration curve and reported in units of nanomoles per milligram of protein [11].

### 3.7 Nitric Oxide Assay (NO)

Tissue samples, weighing approximately 100 mg, were collected from the distal portion of the muscle and skin paddle at the 24-h mark. The measurement of tissue nitric oxide levels was conducted using the Nitric Oxide Assay kit from Abcam, which relies on the Greiss reagent method. The absorbance was recorded spectrophotometrically at a wavelength of 540 nm, and the tissue's nitric oxide content was quantified and reported in micromoles per milligram of tissue [9].

### 3.8 Myeloperoxidase level (MPO)

Tissue-associated MPO level was determined using a method like that described by Hillegas et al. [12] Tissue samples were homogenized in 50 mM potassium phosphate buffer (PB; pH 6.0) and then centrifuged at 41,400 g for 10 min. The resulting pellets were reconstituted in 50 mM PB containing 0.5% hexadecyltrimethylammonium bromide. After subjecting the samples to three freeze-thaw cycles with sonication between cycles, they were once again centrifuged at 41,400 g for 10 min. Subsequently, 0.3 mL aliquots of the prepared samples were combined with a 2.3 mL reaction mixture composed of 50 mM PB, *o*-dianisidine, and a 20 mM H_2_O_2_ solution. One unit of enzyme level was defined as the quantity of MPO that brought about a change in absorbance, measured at 460 nm, over a period of 3 min. MPO level was reported in units per gram of tissue (U/g tissue).

### 3.9 Peripheral neutrophil count

Peripheral blood was collected at the 24-h mark to prepare smears, which were subsequently stained with Wright-Giemsa stain for assessing circulating neutrophils. On each slide, five fields were randomly chosen at high-power-field (200x) magnification, and neutrophil counts were manually conducted. The circulating neutrophil count was computed as 1000 times the average value obtained from these counts [9].

### 3.10 Histopathological analysis

Histopathological analyses were conducted following the methodology as outlined by Ersel et al. [13] Following the fixation of skin biopsy samples in 10% buffered formalin for 24 h, standard paraffin wax embedding procedures were employed and the specimens were sectioned. Sections of approximately 5 μm thickness were obtained using a Leica RM2145 microtome from Germany. These sections were subsequently subjected to staining with both hematoxylin and eosin, as well as Mallory Azan stains. The evaluation of the hematoxylin and eosin- and Mallory Azan-stained sections involved the use of a light microscope (Olympus BX-51 light microscope, Olympus C-5050 digital camera) to assess features such as necrosis, edema, polymorphonuclear leukocyte (PMNL) infiltration and vascularization, employing a modified Verhofstad scoring system [9]. (Table 1). For the immunohistochemical analysis of inducible nitric oxide synthase (iNOS) expression, paraffin sections underwent deparaffinization in xylene overnight to quench endogenous peroxidase activity. Following this, they were immersed in a solution containing 3% H2O2 in methanol. To retrieve antigenicity, the sections were subjected to heat-induced epitope retrieval in a sodium citrate solution within a microwave oven, initially at 90 W for 5 min, followed by 360 W for 15 min. Next, the sections were incubated with the primary antibody (anti-iNOS, 1/100 dilution) for 24 h at 4 °C. Detection of the antibody was accomplished using the Histostain-Plus Bulk Kit from Bioss, Inc., with a secondary antibody specific for rabbit immunoglobulin G. The visualization of the immunoreaction was achieved using 3,3′-diaminobenzidine. The evaluation of immunoreactivity was performed under light microscopy at both ×10 and ×40 magnifications using an Olympus BX-51 light microscope coupled with an Olympus C-5050 digital camera.

**Table 1:** Histopathological scoring system, modified Verhofstad Scoring.

*Score*	Necrosis	Edema	PMNL^a^	Vascularization
0	None	None	Normal	None
1	Superficial	Light	Light	Light
2	Pronounced	Pronounced	Pronounced	Pronounced
3	Massive	Dense	Massive	Dense

a PMNL – polymorph nuclear leukocytes.

## 4 Statistical analysis

Statistical analysis was conducted using Student's T-test to compare two groups. Differences among groups with respect to parameters such as flap survival areas, modified Verhofstad scores, NO, GSH, MPO, MDA levels and tissue neutrophil counts were assessed through one-way ANOVA. In cases where the p-value from the variance analysis indicated statistical significance, post hoc Tukey tests were employed for further comparisons. Results are presented as mean ± standard error of the mean (SEM), and statistical significance was defined as p < 0.05.

## 5 Results

Throughout the study, no fatalities among the rats were recorded, and no complications were observed.

### 5.1 Flap survival area

The flap survival area (23.47 cm^2^) was calculated by subtracting the necrotic region from the total flap area. In the sham group, the mean survival area measured 8.98 ± 1.6 cm^2^, while in the PLC group, it was notably larger at 16.2 ± 2.3 cm^2^. This disparity between the results was found to be statistically significant (p < 0.05).

### 5.2 Biochemical assessments

In the sham group, the mean MDA level was 146.2 ± 12.7 nmol/g, GSH level was 1.4 ± 0.9 nmol/g, MPO was 30.8 ± 5.1 U/g, and NO level was 21.5 ± 6.05 μM/mg of tissue. Conversely, in the PLC group, the mean MDA level was 118.6 ± 11.2 nmol/g, GSH level was 2.3 ± 0.1 nmol/g, MPO was 23.2 ± 4.8 U/g, and NO level was 29.2 ± 9.7 μM/mg of tissue. Notably, statistically significant differences were observed between the two groups across all of these parameters (Table 2).

**Table 2:** Biochemical assesment of mean malondialdehyde (MDA) levels, tissue glutathione (GSH) levels, myeloperoxidase level (MPO) and nitric oxide (NO) of sham and Propionyl-l-carnitine (PLC) group.

*Biochemical Parameters*	Mean MDA (nmol/g)	GSH level (nmol/g)	MPO level (U/g)	NO level (μM/mg)
***Sham group***	146.2 ± 12.7	1.4 ± 0.9	30.8 ± 5.1	21.5 ± 6.05
***PLC group***	*118.6 ± 11.2	*2.3 ± 0.1	*23.2 ± 4.8	*29.2 ± 9.7

*p < 0.05.

### 5.3 Peripheral neutrophil count assessment

In the sham group, the peripheral neutrophil count averaged 23,643 ± 1780 cells/μL, while in the PLC group, it measured 20,425 ± 1355 cells/μL. Significantly, the disparity between these findings was supported by a p-value of <0.05.

### 5.4 Histopathological examination

In an examination of necrosis, edema, PMNL infiltration and vascularization in samples collected from 16 subjects, with eight in each group (PLC and sham), it became evident that the scores for these parameters were in proximity. The average necrosis values for the 16 subjects ranged from 0 to 3, with readings of 2 ± 0.5 for the PLC group and 3.75 ± 0.4 for the sham group. In the sham group, the scores for edema, PMNL and vascularization were 3.62 ± 0.5, 3.1 ± 0.8 and 1 ± 0.9 respectively. On the other hand, the PLC group exhibited mean scores of 1.75 ± 0.4 for edema, 1.87 ± 0.5 for PMNL infiltration, and 2.37 ± 0.7 for vascularization. Consequently, it is apparent that statistically significant differences were identified for all these parameters between the sham and PLC groups (p < 0.05) (Table 3; Fig. 2).

**Table 3:** Histopatological examination of sham and Propionyl-l-carnitine (PLC) group involving necrosis, edema, polymorphonuclear leukocyte (PMNL) infiltration, vascularization.

*Histopatological examination*	Necrosis	Edema	PMNL	Vascularization
***Sham group***	3.75 ± 0.4	3.62 ± 0.5	3.1 ± 0.8	1 ± 0.9
***PLC group***	*2 ± 0.5	*1.75 ± 0.4	*1.87 ± 0.5	*2.37 ± 0.7

*p < 0.05.

## 6 Discussion

The success rate of free tissue applications, a commonly practiced procedure in recent years, typically ranges between 97% and 87%. However, it is noteworthy that partial or complete flap losses in pedicle flaps, along with associated complications can occur at rates as high as 25%. These outcomes are primarily attributed to insufficient perfusion and ischemic damage that can trigger various tissue and vascular alterations which ultimately results flap necrosis [14].

Ischemia/reperfusion injury stands as a pivotal factor significantly influencing flap survival. This intricate process gives rise to the generation of ROS and various harmful substances which in turn trigger processes like apoptosis or necroptosis, leading to cellular damage or demise. A decrease in microcirculation can ultimately result in partial or complete flap necrosis. Antioxidant agents play a crucial role in mitigating the impact of ROS. This includes their ability to counteract oxygen ions, peroxides, free radicals and other byproducts, which tend to exacerbate complement activation, leukocyte-endothelial cell adhesion, platelet-leukocyte aggregation and microvascular permeability during reperfusion following an ischemic period [ [15]. Modern research efforts have delved extensively into the exploration of endogenous and exogenous agents capable of alleviating or diminishing the oxidative damage inflicted by free radicals on crucial cellular components. These components include DNA, proteins, lipids and various other vital constituents [[16], [17], [18], [19]].

l-carnitine plays several vital metabolic roles within the body, with one of the most extensively studied being its function in transporting long-chain fatty acids into the mitochondria's inner compartment. This process is crucial for the β-oxidation reaction and subsequent energy production. Early reports suggest that propionyl-l-carnitine may act by improving myocardial energy metabolism, reducing oxidative stress and enhancing endothelial function. One study found that propionyl-l-carnitine improved glucose oxidation and reduced the intramitochondrial ratio of acetyl-CoA to free CoA, which stimulated the activity of pyruvate dehydrogenase and increased the oxidation of pyruvate [20]. Another study found that propionyl-l-carnitine reduced myocardial injury by counteracting the toxic effect of high levels of free fatty acids, which occur in ischemia, and by improving carbohydrate metabolism [21]. Propionyl-l-carnitine also exerts beneficial effects on fatty acid turnover. It has been observed that the peroxidation of fatty acids in ischemic tissue can lead to the generation of free oxygen radicals, which directly contribute to oxidative damage to cellular macromolecules, including nucleic acids, proteins and lipids. In the 20th century, a notable interest arose in assessing the potential effects of PLC in preventing I/R injuries. Numerous studies in clinical and experimental settings have explored the protective effects of PLC against I/R-induced heart injuries and these investigations have often encompassed other organs as well [4,20,21].

This study aimed to evaluate the impact of PLC on ischemia-reperfusion injury within rat epigastric island flaps, encompassing various macroscopic, biochemical and histopathological parameters. The investigation provides empirical support for the hypothesis that pre-ischemic administration of l-carnitine reduces I/R injury in rat epigastric flaps. To achieve this, we surgically raised 6 × 4 cm epigastric flaps in rats and conducted a comprehensive assessment that included biochemical, histopathological and macroscopic evaluations following a 10-h period of ischemia, consistent with the methodology applied in our prior studies [8,9,14,15].

The designated global ischemia duration for the skin flap was established as 10 h and accordingly, a 10-h ischemic period was implemented in our experimental design. Commencing 24 h post-reperfusion, biochemical analyses were initiated due to the recognition that the levels of oxidant products or end products attain their zenith during the 24-h reperfusion period. Histopathological changes, encompassing angiogenesis, inflammation and edema commenced on the 5th day of the experimental timeline. Consequently, a histopathological assessment was conducted on the 10 t h day to systematically evaluate the temporal progression of observed physiological alterations [3,5,9,14,15]. Biochemical measurements were taken from the right side of the flaps 24 h after reperfusion, while the evaluation of necrotic areas on the left side of the flaps was carried out prior to obtaining samples for histochemical analysis on the tenth postoperative day. This method was employed to enhance the objectivity in quantifying flap necrotic areas. The macroscopic assessment of flap survival areas was performed using the Image-pro express program (Version 6.0, Media Cybernetics, USA). The results showed statistically significant differences in flap survival areas between the PLC group and the sham group.

Detecting the presence of free oxygen radicals has been a challenging task owing to their short half-lives. To address this, we turned to the measurement of MDA, which serves as an indicator of the product of lipid peroxidation within cells. Malondialdehyde levels are known to increase in tandem with the presence of free oxygen radicals in I/R injury. Thus, we utilized MDA measurements as a method to evaluate reperfusion injury [8,15]. Significantly, the PLC group demonstrated a notably lower MDA level in comparison to the sham group. It is important to highlight that glutathione (GSH) plays a critical role as an intracellular antioxidant, effectively countering free radicals and oxygen-derived products [22]. In accordance with consistency, Kurt et al. [23] reported elevated lipid peroxidation and diminished tissue GSH levels in animals subjected to I/R injury.

In the context of this study, it is noteworthy that GSH levels exhibited a significant increase in the PLC group compared to the sham group. Myeloperoxidase is typically found within azurophilic granules in neutrophils and lysosomes in monocytes. The release of MPO from neutrophils during episodes of inflammation can have detrimental effects on cells. MPO has been utilized in certain investigations as a marker for ischemia-reperfusion injury. For instance, Matthijsen et al. [24] established a positive correlation between I/R injury in rats and MPO levels, underscoring its significance in assessing such injuries. Gorur et al. [25] have presented an extensive overview of the results from their experimental study, which focused on the effects of PLC preconditioning on renal ischemia-reperfusion injury in rats. Their research has demonstrated a notable reduction in tissue malondialdehyde, myeloperoxidase and nitric oxide levels in animals that underwent l-carnitine pretreatment before the induction of renal ischemia-reperfusion injury, as compared to the control group.

Nitric oxide plays a vital role by exerting antioxidative properties through the inhibition of leukocyte activation and by enhancing superoxide anion production [26]. It is important to note that ROS are recognized for their ability to reduce NO levels [8]. In a study conducted by Lille et al. [27], a reduction in muscle-flap ischemia-reperfusion injury was observed, accompanied by an elevation in cyclic adenosine monophosphate and NO levels. Additionally, Khiabani et al. [28] reported a positive correlation between the survival of random skin flaps and NO levels. In the present study, the PLC group demonstrated higher NO levels in comparison to the sham group.

Histopathological assessment revealed a substantial increase in inflammation and reperfusion injury parameters, including necrosis, polymorphonuclear leukocyte (PMNL) infiltration, and edema, within the Sham group. Conversely, the PLC group displayed reduced scores across these categories, indicating the robust ability of PLC to prevent reperfusion injury through its anti-inflammatory effects. Notably, the PLC group also exhibited an evident enhancement in vascularity, suggesting that PLC promotes increased blood supply, thereby ameliorating the effects of ischemia. Neutrophils play a role in impeding tissue perfusion due to capillary obstruction and an elevated neutrophil count is a common characteristic of ischemia-reperfusion injury [ [8,9]]. The reduction in neutrophil count observed in the PLC group signifies a beneficial impact on I/R injury. This reduction in neutrophil count is indicative of a favorable outcome, suggesting that PLC exerts a protective effect against I/R injury by modulating the neutrophil response.

While multiple prior studies have indeed highlighted the impact of PLC on I/R damage, it is worth noting that, to the best of our knowledge, this study represents the first all-encompassing examination of its effectiveness at macroscopic, biochemical, and histological levels. However, it is important to acknowledge certain limitations within the current investigation. Firstly, the sample size was relatively small. Secondly, the assessment was confined to macroscopic necrotic areas and the evaluation of histological and biochemical markers. Subsequent research is imperative to provide further validation of the innovative therapeutic potential of PLC in the realm of I/R injuries.

## 7 Conclusion

In summary, this study underscores the prospective clinical utility of Propionyl-l-carnitine in mitigating the adverse consequences of I/R injury. This application is especially pertinent in the realm of reconstructive surgery, where tissue flaps are frequently employed.

## Ethical approval

This study is approved by Demiroglu Science University Ethical Committee as study number #1217015402. All procedures performed in accordance with the ethical standards of the institutional and/or national research committee.

## Funding

No funding was received.

## Additional information

No additional information is available for this paper.

## 8 Data availability statement

There is no research related data stored in publicly available repositories, and the data will be made available on request.

## CRediT authorship contribution statement

**Atilla Adnan Eyuboglu:** Writing – review & editing, Writing – original draft, Visualization, Validation, Supervision, Methodology, Investigation, Funding acquisition. **Ovunc Akdemir:** Writing – original draft, Visualization, Validation, Software, Resources, Project administration, Methodology, Investigation, Funding acquisition, Formal analysis, Data curation, Conceptualization. **Oytun Erbas:** Writing – review & editing, Visualization, Software, Methodology. **Mustafa Tonguc Isken:** Writing – review & editing, Visualization, Supervision. **Feng Zhang:** Supervision, Software, Investigation, Formal analysis, Data curation, Conceptualization. **William C. Lineaweaver:** Visualization, Project administration, Methodology, Formal analysis, Data curation, Conceptualization.

## Declaration of competing interest

The authors declare that they have no known competing financial interests or personal relationships that could have appeared to influence the work reported in this paper.
